# Safety of Prescription Nonsteroidal Anti‐inflammatory Drugs in Adults With Inflammatory Bowel Disease: Data From a Large Administrative Claims Cohort

**DOI:** 10.1002/acr.80067

**Published:** 2026-05-05

**Authors:** Adam S. Mayer, Rui Xiao, Meenakshi Bewtra, Michael D. George, Pamela F. Weiss

**Affiliations:** ^1^ Center for Discovery and Innovation, Hackensack Meridian Health Nutley New Jersey; ^2^ Hackensack University Medical Center Hackensack New Jersey; ^3^ University of Pennsylvania Philadelphia; ^4^ The Children's Hospital of Philadelphia Philadelphia Pennsylvania

## Abstract

**Objective:**

The concern that nonsteroidal anti‐inflammatory drugs (NSAIDs) may precipitate flares of inflammatory bowel disease (IBD) has limited their use in managing musculoskeletal symptoms in those with IBD, but safety data are mixed.

**Methods:**

This retrospective cohort study included patients with IBD aged at least 18 years from Optum's deidentified Clinformatics Data Mart Database (2000–2022). Patients with a new NSAID prescription fill were matched to those without an NSAID fill during the study period. The index date for exposed patients was the date of first NSAID fill, with a matched date for unexposed. Propensity score‐based inverse probability of treatment‐weighted Cox proportional hazards models evaluated the association between NSAID exposure and time to IBD‐related hospitalization across IBD subtypes. A hazard ratio (HR) of 1.2 was prespecified as the non‐inferiority margin.

**Results:**

Among 348,095 patients with IBD, 23.2% were NSAID‐exposed. NSAID exposure was associated with a small increase in IBD‐related hospitalization in the overall cohort but demonstrated non‐inferiority (HR 1.07, 95% confidence interval [CI] 1.04–1.10). Non‐inferiority was met with no significantly increased risk of IBD‐related hospitalization in patients with ulcerative colitis (HR 0.97, 95% CI 0.93–1.02). In contrast, non‐inferiority was not met in the Crohn disease subgroup (HR 1.16, 95% CI 1.12–1.21).

**Conclusion:**

Patients with ulcerative colitis did not have a significantly increased risk of IBD‐related hospitalization associated with NSAID exposure and met non‐inferiority, whereas non‐inferiority could not be established for those with Crohn disease. These results suggest that NSAID use may be acceptable for select patients with IBD who have significant musculoskeletal symptoms, especially those with ulcerative colitis.

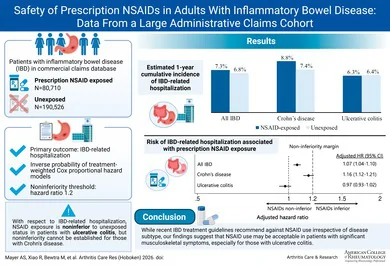

## INTRODUCTION

Musculoskeletal symptoms are common in patients with inflammatory bowel disease (IBD), with inflammatory arthritis or enthesitis present in up to half of cases.^1^, ^2^, ^3^ Nonsteroidal anti‐inflammatory drugs (NSAIDs) are commonly used in the treatment of both inflammatory and noninflammatory musculoskeletal symptoms because of their effectiveness, low cost, and ease of access. However, there is a historical concern that NSAIDs may precipitate intestinal disease flares in patients with IBD, largely based on data from small, single‐center studies. A 2006 prospective cohort study of 119 patients with quiescent IBD, for example, found that 17% to 28% of patients exposed to nonselective NSAIDs experienced clinical relapse compared with none of the patients exposed to acetaminophen.^4^

**SIGNIFICANCE & INNOVATIONS**

- In a large propensity score‐weighted study of adult patients with inflammatory bowel disease (IBD), nonsteroidal anti‐inflammatory drug (NSAID) exposure was not associated with a meaningful increase in IBD‐related hospitalization in patients with ulcerative colitis, whereas similar conclusions could not be definitively reached in those with Crohn disease.
- Over half of NSAID‐exposed individuals had a recent code for joint or chronic musculoskeletal pain, suggesting that musculoskeletal symptoms are an important indication for NSAID prescription in patients with IBD.
- Although recent IBD treatment guidelines recommend against NSAID use, irrespective of disease subtype, our findings suggest that NSAID use may be acceptable in patients with significant musculoskeletal symptoms, especially for those with ulcerative colitis.

However, the literature on NSAID safety in IBD has been mixed. There is concern that previous studies attributing risk to NSAID exposure have suffered from residual confounding and reverse causality.^5^ Moreover, the small sample sizes of many previous studies have limited statistical power and the ability to conduct subgroup analyses. More recent data suggest that the risk of IBD flare may depend on IBD subpopulation and NSAID formulation. A large 2016 cohort study of patients with self‐reported IBD in remission found an increased risk of relapse (relative risk [RR] 1.65, 95% CI 1.12–2.44) in those with Crohn disease (CD) subtype who reported at least five NSAID doses per month compared with those with lower intake, but no risk was identified in those with ulcerative colitis (UC).^6^ In a 2018 meta‐analysis of NSAID safety studies at low risk of bias, there was no significant increase in risk associated with NSAID exposure in patients with UC (pooled RR 0.94 [95% CI 0.36–2.42]) in contrast to the CD subgroup (pooled RR 1.53 [1.08–2.16]).^7^ Additionally, nonselective NSAIDs pose a greater risk of gastrointestinal toxicity than cyclo‐oxygenase–2 (COX‐2) selective NSAIDs because of the decrease in mucosal prostaglandins from COX‐1 inhibition that leads to decreased mucus secretion and mucosal blood flow and thus predisposes to mucosal injury.^8^, ^9^, ^10^ There have been two small randomized, placebo‐controlled trials of COX‐2 selective inhibitors that have not shown an increased associated risk of IBD flare,^11^, ^12^ but safety has not been confirmed in larger real‐world patient cohorts.

Although the risk associated with NSAID exposure in patients with IBD is inconclusive, there are known analgesic benefits in patients with musculoskeletal symptoms. Therefore, it is reasonable to hypothesize that there are certain subgroups of patients with IBD for whom there may be a threshold of acceptable risk below which NSAID exposure may be non‐inferior to a lack of exposure. However, the concerns for IBD flare risk have prompted the recent 2018/2019 American College of Gastroenterology (ACG) guidelines for both CD and UC to make strong recommendations against NSAID use irrespective of disease subtype and without distinguishing between NSAID formulations.^13^, ^14^ These recommendations leave providers and patients with imperfect alternatives for analgesia, such as opioids or increased immunosuppression. As such, we leveraged a large administrative claims cohort to systematically evaluate the association of severe IBD flares with prescription NSAID exposure across IBD subtypes and NSAID formulations, using advanced propensity score methods to address confounding within a non‐inferiority design.

## MATERIALS AND METHODS

The protocol for the conduct of this study was reviewed by the Children's Hospital of Philadelphia Institutional Review Board (IRB) and determined to be not human subject research (IRB #23–020917, March 27, 2023); there was therefore no patient or public involvement. All procedures were performed in compliance with relevant laws and institutional guidelines.

### Data source

This retrospective cohort study used data from January 2000 to June 2022 in Optum's deidentified Clinformatics Data Mart Database (Optum CDM), a national database of administrative health claims for members of commercial and Medicare Advantage health plans. Available data included deidentified patient characteristics, *International Classification of Diseases* (*ICD*)*‐9* and *ICD‐10* diagnosis codes, National Drug Codes (NDC) and Healthcare Common Procedure Coding System (HCPCS) codes for medications, prescription fill dates, emergency department (ER) and hospitalization dates, and Current Procedural Terminology (CPT) codes for gastrointestinal surgical procedures.

### Patient population

Adult (aged ≥18 years) patients with IBD were included. A diagnosis of IBD was defined based on validated algorithms^15^, ^16^, ^17^, ^18^, ^19^, ^20^ and required all of the following: 1) at least two inpatient or outpatient diagnosis codes of CD (ICD‐9: 556.xx/ICD‐10: K51.xx) and/or UC (ICD‐9: 555.xx/ICD‐10: K50.xx) at least 30 days apart, 2) at least one diagnosis code from an outpatient encounter, and 3) a minimum of six months of continuous enrollment. Patients without pharmacy claims data were excluded. For IBD type, a patient was assigned a diagnosis of CD or UC if at least 75% of IBD codes from the time of Optum CDM enrollment to the index date were CD‐ or UC‐specific codes, respectively. A patient was labeled as indeterminate colitis (IC) if they did not meet this majority threshold for CD or UC codes.

### Exposure

The primary exposure was a new prescription NSAID fill (Supplementary Table 1) with a six‐month look‐back period. Aspirin was not included in the exposure definition to minimize heterogeneity of exposure effect.^5^, ^7^ For the exposed group, only the first eligible NSAID exposure was included. Patients without an NSAID exposure during the study period were considered unexposed.

### Index date

The index date for exposed patients was the date of first NSAID fill (Figure 1). Each exposed patient was matched to two to three unexposed patients by age (within five years) and calendar year during which IBD criteria were met (within one year). Unexposed patients were assigned an index date matched for time from IBD diagnosis to index date in the exposed counterpart (Supplementary Figure 1).

**Figure 1:**
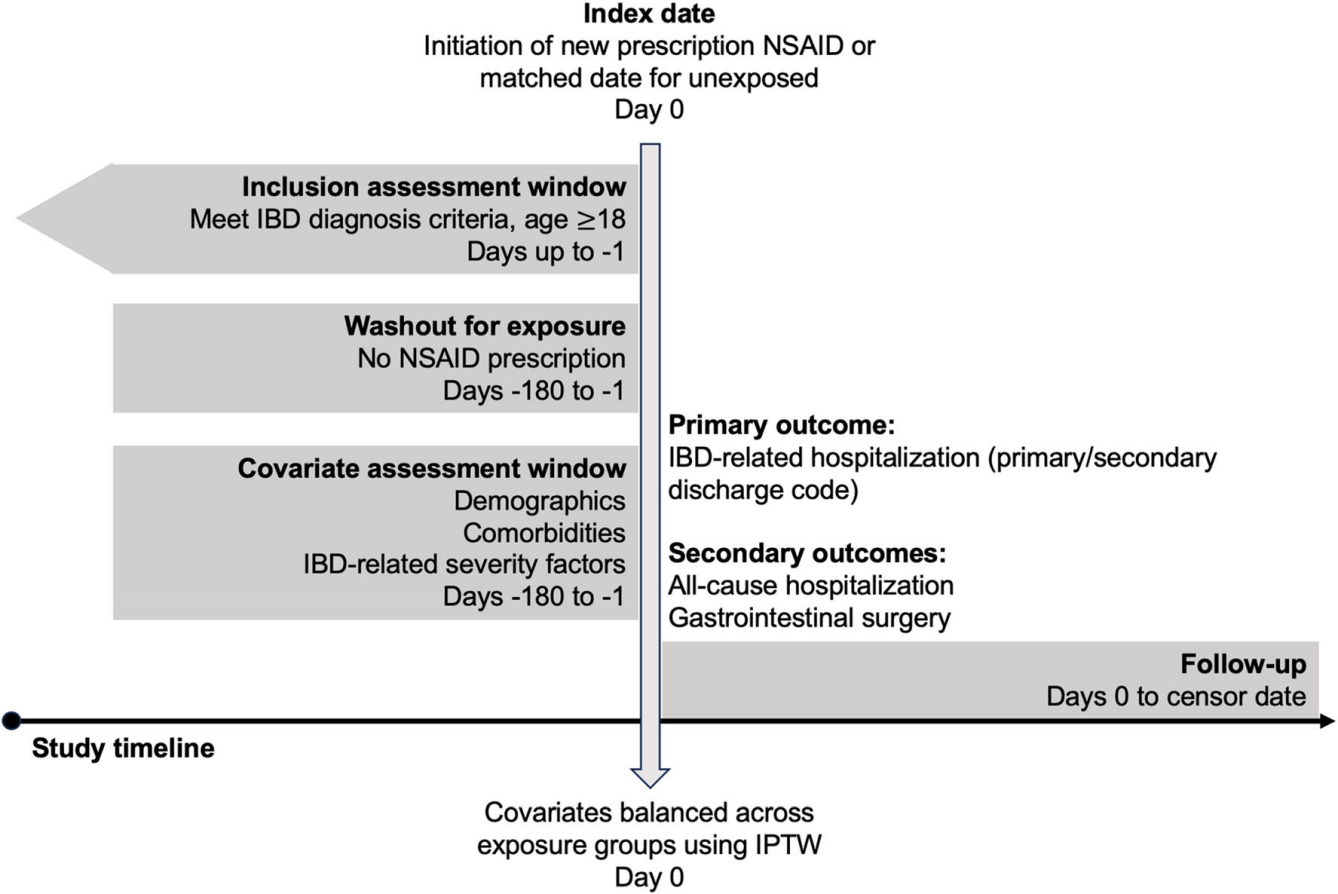
Overview of study design. Participants were censored at the end of continuous enrollment, end of study period, or at least six months from last NSAID fill. Cox PH models for primary and secondary outcomes were adjusted for covariates that did not achieve balance by propensity scores. IBD, inflammatory bowel disease; IPTW, inverse probability of treatment weighting; NSAID, nonsteroidal anti‐inflammatory drug; PH, proportional hazards.

### Patient follow‐up

The follow‐up period was defined as the index date until whichever of the following occurred first: 1) end of study period, 2) end of continuous enrollment in Optum CDM, 3) occurrence of outcome of interest, or 4) end of “continuous” NSAID exposure. NSAID exposure was considered “continuous” as long as subsequent NSAID prescriptions were filled within six months of the preceding fill date. NSAID exposure ended six months or more from the last fill date.

### Outcomes

The primary outcome was IBD‐related hospitalization, which was defined as any inpatient hospitalization with a duration of more than one day with an IBD diagnosis code as the primary or secondary discharge code. A length of stay of more than one day was required to exclude admissions for elective endoscopic procedures or biologic infusions.^21^ Secondary discharge codes were included in the outcome definition to capture instances in which IBD‐related symptom codes, such as abdominal pain or gastrointestinal bleeding, were listed as the primary discharge code, and IBD was the secondary code for an IBD‐related hospitalization. Secondary outcomes included all‐cause hospitalization and gastrointestinal surgery.

### Statistical analysis

Differences in characteristics between NSAID‐exposed and unexposed patients were summarized using descriptive statistics and compared with standardized mean differences (SMD) and two‐sample *t*‐tests. Propensity scores for NSAID exposure were estimated using multivariable logistic regression, modeling the probability of treatment assignment based on observed baseline characteristics to reduce confounding between exposure groups.^22^ The resulted propensity scores were used to create stabilized inverse probability of treatment weights (IPTW) to balance confounders across exposure groups.^22^, ^23^ Covariate balance between exposure groups was assessed pre‐ and post‐IPTW using SMDs, with an SMD of less than 0.1 considered to be adequate balance.^24^ Any unbalanced covariates were adjusted for separately in the final model.

IPTW‐weighted Cox proportional hazards (PH) models evaluated the association between NSAID exposure and time to each primary and secondary outcome across IBD subtypes. Robust SEs were used to avoid potential nonconstant variance of errors (heteroscedasticity).^25^ Separate propensity scores and Cox PH models were developed for the overall IBD cohort and the CD and UC subgroup analyses. A hazard ratio (HR) of 1.2 was prespecified as the non‐inferiority margin based on discussions with IBD content experts on what magnitude of increased risk of IBD‐related hospitalization (compared with baseline risk in the unexposed) would be clinically acceptable in light of the potential benefits of NSAID use. The PH assumption for the primary analysis was assessed with Schoenfeld residuals and visual examination of log‐log plots. To determine absolute risk and the number needed to harm (NNH) from NSAID exposure, predicted one‐year cumulative incidence rates were calculated using linear predictions from the Cox PH models.

Given the expected sample size of over 80,000 NSAID‐exposed patients and an estimated annual IBD‐related hospitalization incidence rate of 5% to 10%, the power to detect a HR within the prespecified non‐inferiority margin is over 99%. All statistical analyses were performed using Stata version 18.0 (College Station, TX, USA).

### Covariates

Covariates were measured during a six‐month period before the index date and defined by corresponding ICD, NDC, HCPCS, or CPT codes (Supplementary Tables 2 and 3). These covariates included continuous/ordinal variables (age at the index date, calendar year of the index date, and Charlson comorbidity index) and binary variables (biologic sex, baseline cardiovascular disease, peripheral vascular disease, chronic kidney or liver disease, joint pain, chronic musculoskeletal pain, depression, anxiety, psychotic disorders, menstrual disorders, all‐cause hospitalization, gastrointestinal bleed, gastrointestinal surgery, anal fistula, intestinal fistula, intestinal stricture, gastrointestinal abscess, anticoagulant/antiplatelet prescription fill, opioid fill, glucocorticoid fill, conventional synthetic disease‐modifying antirheumatic drug [csDMARD] fill, or advanced DMARD fill). Targeted synthetic DMARDs (tsDMARDs) were combined with biologic DMARDs (bDMARDs) into a category of advanced DMARD therapies because tsDMARDs were first introduced to the US market more than halfway through the study period and used by only a small minority of patients in this study.

The IBD severity index, validated in Optum CDM for the severity of underlying IBD, incorporates factors such as weight loss, anemia, glucocorticoid use, and intestinal disease location and is independently associated with the risk of IBD‐related hospitalization.^26^ Separate indices have been validated for CD and UC, but no validated index exists for those with IC. As such, IBD severity indices were adjusted for separately in the CD and UC Cox PH analyses and not included in the propensity score models.

For each patient, the absence of a given ICD, NDC, HCPCS, or CPT code was assumed to indicate the absence of the corresponding diagnosis, prescription, or procedure. To our knowledge, no other data were missing beyond the inherent risk of miscoding in claims databases.

### Sensitivity analyses

Several predefined sensitivity analyses were performed for the primary analysis in the overall IBD cohort using separate propensity score models. Specific subpopulations examined included those with COX‐2 selective NSAID exposure at the index date, those with repeated NSAID use (defined as ≥2 NSAID fills within six months of the index date), and participants with baseline immunosuppression use (≥1 fill for a csDMARD, bDMARD, or tsDMARD during the baseline period). Different definitions of the primary outcome were also examined, including 1) hospitalizations with an IBD code in the primary position only, 2) IBD‐related hospitalization or IBD‐related ER visit (IBD code in primary or secondary position for either), and 3) IBD‐related hospitalization within 12 months of the index date. A final sensitivity analysis truncated the IPTW at the 1st and 99th percentiles to remove extreme outlier weights.^27^

## RESULTS

### Cohort characteristics

Among the 348,095 patients with IBD identified in Optum CDM, 80,710 NSAID‐exposed and 190,526 matched unexposed patients were included. Before weighting, exposed patients had a significantly higher prevalence of baseline chronic musculoskeletal pain (SMD 0.39), joint pain (SMD 0.30), glucocorticoid use (SMD 0.20), opioid use (SMD 0.31), and all‐cause hospitalization in the past six months (SMD 0.13; *P* < 0.01 for all) (Table 1) compared with unexposed individuals. All covariates were adequately balanced across groups after IPTW (Table 1, Supplementary Figures 2 and 3). Exposed patients had a median of two NSAID prescription fills (interquartile range [IQR] 1–4), with 53.4% having at least two fills during the study period. The median days supplied for the index NSAID prescription was 20 days (IQR 10–30). A total of 12.8% of exposed patients received a COX‐2 selective NSAID. Among those with repeated NSAID use, there was a median of three fills (IQR 2–7).

**Table 1:** Baseline characteristics of participants, stratified by NSAID exposure*

Demographics	NSAID‐exposed (N = 80,710), n (%)	Unexposed (N = 190,526), n (%)	SMD pre‐weight	SMD post‐weight
Age at index date, mean (SD)	52.2 (16.1)	52.2 (17.0)	0.003	0.003
Female	48,018 (59.5)	108,321 (56.9)	0.054	0.000
Follow‐up time, median (IQR), y	0.6 (0.5–2.1)	1.2 (0.4–3.1)	0.132	–
IBD features				
IBD subtype			0.044	–
CD	33,739 (41.8)	81,694 (42.9)		
UC	35,185 (43.6)	83,863 (44.0)		
Indeterminate colitis	11,786 (14.6)	24,969 (13.1)		
Anal fistula	2,113 (2.6)	4,519 (2.4)	0.016	0.001
Intestinal fistula	1,217 (1.5)	2,996 (1.6)	0.005	0.000
Abscess	3,105 (3.8)	6,711 (3.5)	0.017	0.001
Intestinal stricture	5,406 (6.7)	11,551 (6.1)	0.026	0.004
CD severity index, median (IQR)	3 (1–5)	2 (1–4)	0.081	–
UC severity index, median (IQR)	3 (0–6)	3 (0–6)	0.022	–
Other clinical features				
CCI, median (IQR)	0 (0–2)	0 (0–1)	0.003	0.009
Joint pain	40,077 (49.7)	66,985 (35.2)	0.297	0.000
Chronic pain	45,227 (56.0)	70,433 (37.0)	0.389	0.003
Gastrointestinal bleed	22,314 (27.6)	45,095 (23.7)	0.091	0.002
Depression	12,307 (15.2)	21,919 (11.5)	0.110	0.006
Anxiety	11,250 (13.9)	20,062 (10.5)	0.104	0.004
Psychotic disorder	2,482 (3.1)	3,684 (1.9)	0.073	0.002
Menstrual disorder	2,531 (3.1)	3,374 (1.8)	0.088	0.001
At least one all‐cause hospitalization	17,526 (21.7)	31,630 (16.6)	0.130	0.015
Medications				
NSAID type (index date)				
Nonselective	70,372 (87.2)	–	–	–
COX‐2 selective	10,338 (12.8)	–	–	–
Anticoagulation/antiplatelets	3,567 (4.4)	10,135 (5.3)	0.042	0.005
Opioids	27,058 (33.5)	38,107 (20.0)	0.309	0.003
Glucocorticoids	28,198 (34.9)	49,022 (25.7)	0.201	0.004
Other immunosuppression				
csDMARD	33,450 (41.4)	78,579 (41.2)	0.004	0.008
bDMARD/tsDMARD	11,513 (14.3)	28,273 (14.8)	0.016	0.005

* All covariates were measured in the six months before index date. Horizontal dashed lines indicate that covariate was not included in the propensity score model. bDMARD, biologic disease‐modifying antirheumatic drug; CCI, Charlson Comorbidity Index; CD, Crohn disease; COX‐2, cyclo‐oxygenase–2; csDMARD, conventional synthetic DMARD; IBD, inflammatory bowel disease; IQR, interquartile range; NSAID, nonsteroidal anti‐inflammatory drug; SMD, standardized mean difference (≤0.1 is adequately balanced); tsDMARD, targeted synthetic DMARD; UC, ulcerative colitis.

### Rate of IBD‐related hospitalization

A total of 10.1% of exposed patients had an IBD‐related hospitalization compared with 9.6% in the unexposed group. In the unweighted analyses, NSAID‐exposed participants experienced 6.1 IBD‐related hospitalizations per 100 person‐years compared with 4.7 events per 100 person‐years in the unexposed group (Figure 2, Table 2, Supplementary Table 4). After IPTW‐weighting, the estimated incidence of IBD‐related hospitalization in the NSAID‐exposed remained higher than in unexposed individuals (5.6 vs 4.9 events/100 person‐years). In the Cox PH model for the overall IBD cohort, NSAID exposure was associated with an increased risk of IBD‐related hospitalization, but the upper limit of the CI was below the prespecified non‐inferiority margin of 1.20 (HR 1.07, 95% CI 1.04–1.10) (Figure 3), meeting criteria for non‐inferiority. In subgroup analyses, NSAID exposure was non‐inferior in patients with UC (HR 0.97, 95% CI 0.93–1.02) but not in those with CD (HR 1.16, 95% CI 1.12–1.21). Visual inspection of log‐log plots for the primary analysis showed no evidence of violation of the PH assumption (Supplementary Figure 4).

**Figure 2:**
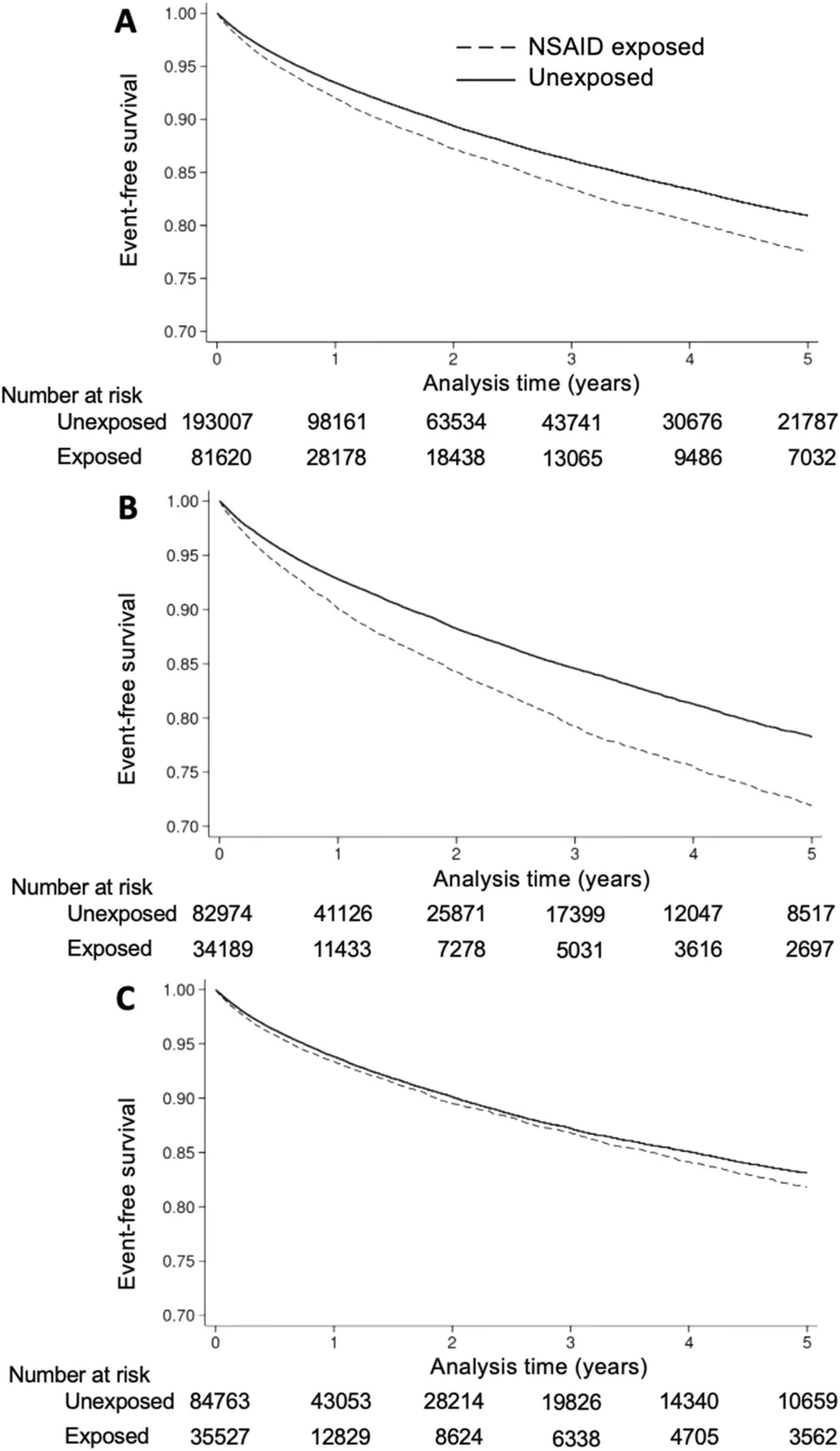
Unweighted Kaplan‐Meier curve for IBD‐related hospitalization in patients with inflammatory bowel disease (A), Crohn disease (B), and ulcerative colitis (C). NSAID, nonsteroidal anti‐inflammatory drug.

**Table 2:** Unadjusted rates of IBD‐related hospitalization, all‐cause hospitalization, and gastrointestinal surgery across IBD subgroups*

Outcome	NSAID‐exposed	Unexposed
IBD‐related hospitalization		
All IBD		
Number of patients	80,710	190,526
Patient‐years	134,853	390,594
Events	8,161	18,203
Incidence rate per 100 patient‐years	6.1	4.7
CD		
Number of patients	33,739	81,694
Patient‐years	53,796	159,931
Events	4,187	8.640
Incidence rate per 100 patient‐years	7.8	5.4
UC		
Number of patients	35,185	83,863
Patient‐years	63,314	178,752
Events	2,999	7,292
Incidence rate per 100 patient‐years	4.7	4.1
All‐cause hospitalization		
All IBD		
Number of patients	80,710	190,526
Patient‐years	91,753	315,500
Events	26,891	53,914
Incidence rate per 100 patient‐years	29.3	17.1
CD		
Number of patients	33,739	81,694
Patient‐years	37,228	130,386
Events	11,530	23,302
Incidence rate per 100 patient‐years	31.0	17.9
UC		
Number of patients	35,185	83,863
Patient‐years	42,006	142,472
Events	11,767	23,630
Incidence rate per 100 patient‐years	28.0	16.6
Gastrointestinal surgery		
All IBD		
Number of patients	80,710	190,526
Patient‐years	140,939	407,226
Events	4,885	9,911
Incidence rate per 100 patient‐years	3.5	2.4
CD		
Number of patients	33,739	81,694
Patient‐years	58,435	169,896
Events	2,193	4,398
Incidence rate per 100 patient‐years	3.8	2.6
UC		
Number of patients	35,185	83,863
Patient‐years	64,350	183,762
Events	2,077	4,227
Incidence rate per 100 patient‐years	3.2	2.3

* CD, Crohn disease; IBD, inflammatory bowel disease; NSAID, nonsteroidal anti‐inflammatory drug; UC, ulcerative colitis.

**Figure 3:**
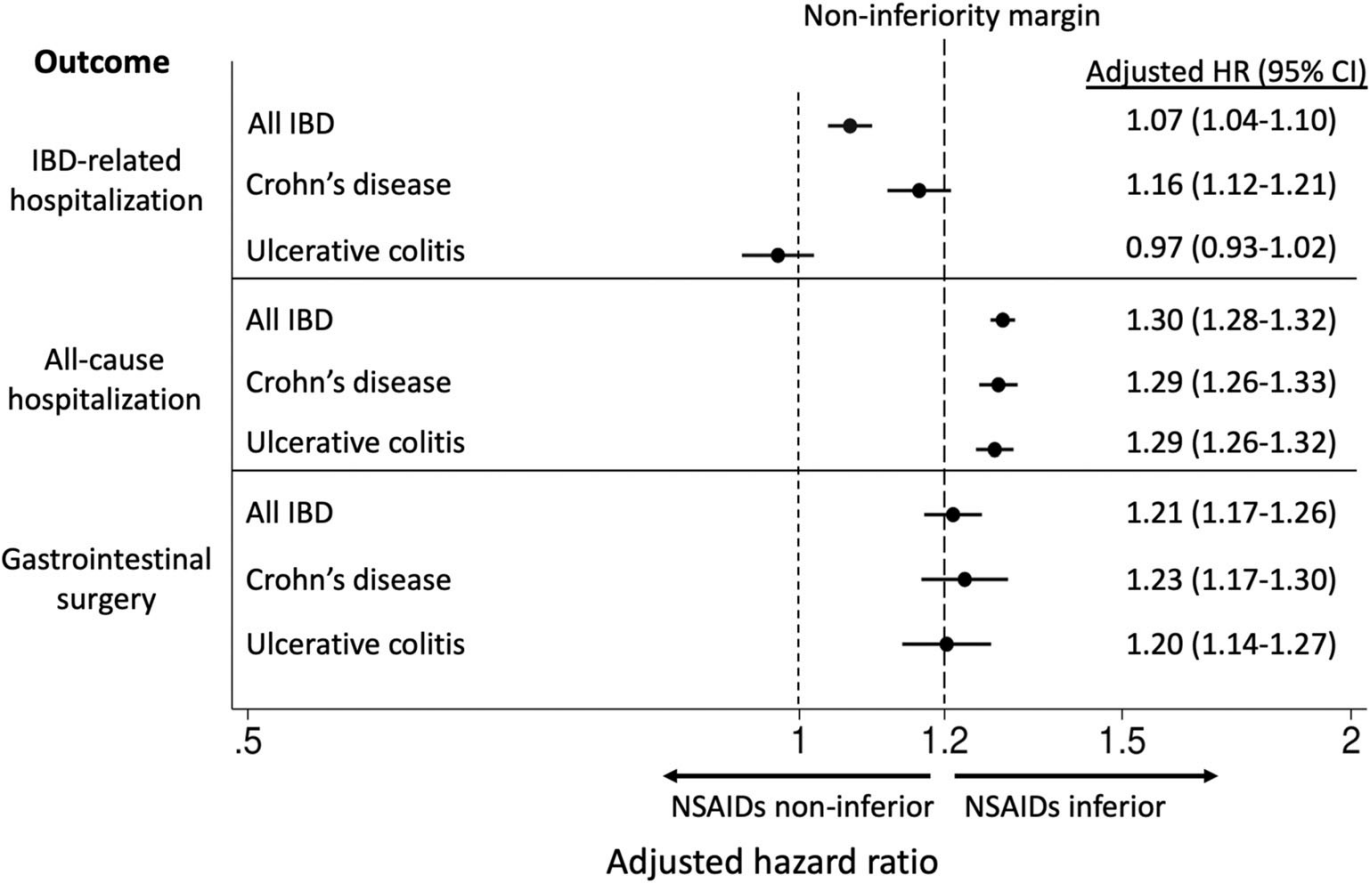
Association between prescription NSAID exposure and time to IBD‐related hospitalization, all‐cause hospitalization, and gastrointestinal surgery across IBD subtypes. Vertical dashed lines indicate 1) null HR 1.0 and 2) the prespecified non‐inferiority margin of HR 1.2 based on clinically‐acceptable risk defined by IBD content experts. *x*‐axis is log‐transformed. CI, confidence interval; HR, hazard ratio; IBD, inflammatory bowel disease; NSAID, nonsteroidal anti‐inflammatory drug.

Table 3 demonstrates the absolute risk of IBD‐related hospitalization in both groups. The NNH attributable to one year of NSAID exposure was 200 (95% CI 143–333) in the overall IBD cohort, 71.4 (95% CI 56–100) for those with CD, and N/A (95% CI 333–∞) in the UC subgroup.

**Table 3:** Estimated one‐year absolute risk of IBD‐related hospitalization*

Group	Adjusted 1‐year cumulative incidence rate	Rate difference (95% CI)	NNH (95% CI)
All IBD			
NSAID‐exposed	7.3%	0.5 (0.3–0.7)	200 (143–333)
Unexposed	6.8%		
CD			
NSAID‐exposed	8.8%	1.4 (1.0–1.8)	71 (56–100)
Unexposed	7.4%		
UC			
NSAID‐exposed	6.3%	−0.1 (−0.4 to 0.2)	N/A (333–∞)
Unexposed	6.4%		

* Cumulative incidence rates calculated from linear predictors of Cox PH models. CD, Crohn disease; CI, confidence interval; IBD: inflammatory bowel disease; NNH, number needed to harm; PH, proportional hazard; UC, ulcerative colitis.

### Rate of all‐cause hospitalization

A total of 33.3% of NSAID‐exposed patients had an all‐cause hospitalization during the study period versus 28.3% of unexposed patients (Table 2). After IPTW‐weighting, the adjusted incidence rate was 27.1 events per 100 person‐years in the exposed versus 18.0 events per 100 person‐years in the unexposed. NSAID exposure was associated with a significantly increased risk of all‐cause hospitalization above the prespecified non‐inferiority margin in allcomers with IBD (HR 1.29, 95% CI 1.27–1.31), as well as the CD (HR 1.28, 95% CI 1.25–1.32) and UC (HR 1.28, 95% CI 1.25–1.31) subgroups (Figure 3).

### Rate of gastrointestinal surgery

A total of 6.1% of NSAID‐exposed patients underwent gastrointestinal surgery compared with 5.2% of unexposed patients (Table 2). The adjusted incidence rate in the exposed group was 3.3 surgeries per 100 person‐years compared with 2.5 surgeries per 100 person‐years in the unexposed group. NSAID exposure was associated with an increased risk of gastrointestinal surgery in the overall IBD cohort, with a 95% CI that crossed the non‐inferiority margin (HR 1.21, 95% CI 1.17–1.26) (Figure 3). Findings were similar in those with CD (HR 1.23, 95% CI 1.17–1.30) and UC (HR 1.20, 95% CI 1.14–1.27).

### Sensitivity analyses

The results of the prespecified sensitivity analyses did not differ from those of the primary analysis in the overall IBD cohort. NSAID exposure retained non‐inferiority for both those with nonselective (HR 1.06, 95% CI 1.03–1.09) or COX‐2 selective NSAID exposure at the index date (HR 1.09, 95% CI 1.01–1.18), repeated NSAID exposure (HR 1.05, 95% CI 1.01–1.08), or baseline immunosuppression (HR 1.11, 95% CI 1.07–1.15). In subgroup sensitivity analyses, conclusions of non‐inferiority did not differ from the primary analysis for either CD or UC when examining across COX‐selectivity or among those with repeated NSAID use. Similar findings were observed with IBD‐related hospitalizations within 12 months of the index date (HR 1.03, 95% CI 0.99–1.07), outcome definitions based on the primary hospitalization discharge code (HR 0.97, 95% CI 0.93–1.01), or with IBD‐related hospitalizations or ER visits (HR 1.07, 95% CI 1.04–1.10). Truncation of the IPTW did not alter the results of the primary analysis.

## DISCUSSION

This is a large propensity score‐weighted administrative claims study in which exposure to prescription NSAIDs was found to be non‐inferior to unexposed patients with respect to IBD‐related hospitalization as a proxy for severe IBD flares. When assessed by IBD subtype, patients with UC did not have a significantly increased risk of IBD‐related hospitalization and met non‐inferiority, whereas non‐inferiority could not be established for those with CD. These findings were robust as evidenced by the consistent results across numerous sensitivity analyses. Although further prospective studies are needed, these results suggest that the risk of NSAIDs may be tolerable in select patients with IBD with significant musculoskeletal symptoms, especially among those with UC.

Reassuringly, the risk estimates observed in the overall IBD cohort in this study are consistent with the pooled risk estimates from a recent meta‐analysis addressing NSAID safety in IBD, but with the current study including a significantly larger sample size than any of the studies included in the meta‐analysis.^7^ A 2022 study in the Veterans Affairs Corporate Data Warehouse was the only other large claims study to assess NSAID safety, and that study did not stratify the analysis by IBD subtype.^5^ The risk of IBD‐related hospitalization observed in that cohort was higher (HR 1.24, 95% CI 1.16–1.33) than that in the current study, although a different outcome definition was used and the nearly all‐male veteran population likely represents a higher‐risk population with a greater burden of comorbidities compared with the commercially‐insured population in Optum CDM. The more extensive balance of confounders in our study may further explain the lower risk estimates compared with the Veterans Affairs cohort.

The enhanced risk of IBD flare associated with NSAID exposure in those with CD relative to UC reported herein is in accordance with previous studies.^6^, ^7^ It remains unclear why patients with CD may be at higher risk from NSAID exposure than those with UC, but the broader anatomic involvement of CD, including the upper gastrointestinal tract where NSAID toxicity is most common,^28^ is one possible cause. Furthermore, a dose‐response relationship of NSAID toxicity has been demonstrated in patients with CD.^6^, ^29^ NSAID dose, however, was not examined in our study because of the inability to confirm consumed dosage over time or to reliably equate doses across NSAID formulations in claims databases.

Non‐inferiority was similarly concluded for those patients with IBD and COX‐2 selective NSAID exposure despite the wider CIs related to the relatively smaller sample size in this subgroup. These results in our cohort of over 10,000 COX‐2 selective NSAID‐exposed patients support the findings of the small observational, open‐label, and randomized clinical trials, which have not consistently found an increased risk for IBD relapse attributable to COX‐2 selective NSAIDs.^4^, ^11^, ^12^, ^30^, ^31^ Ultimately, this study's results suggest that there may be more nuance to the risk of NSAID use in this population than declared in the recent ACG IBD treatment guidelines. The findings from the current study support the 2024 European Crohn's and Colitis Organization guidelines that recommend a case‐by‐case determination of the risk/benefit of NSAID use in patients with IBD and arthropathy, specifically noting that risk may be lower in those with UC and when using COX‐2 selective inhibitors for short periods of time.^32^

The overall composition of the cohort in this study, with approximately equal proportions of patients with CD and UC and a minority IC subgroup, is consistent with other large published IBD cohorts.^5^, ^33^ The proportion of patients who were prescribed NSAIDs is also similar to that observed in other IBD cohorts.^5^ As expected, patients with NSAID exposure had a higher baseline use of other analgesics and anti‐inflammatory agents, such as opioids and glucocorticoids, as well as higher baseline rates of all‐cause hospitalization, but reassuringly these covariates were all adequately balanced using IPTW. Over half of the NSAID‐exposed group had at least one code for joint or chronic musculoskeletal pain at baseline, suggesting that musculoskeletal symptoms are an important indication for NSAID prescription in this cohort, thus reinforcing the relevance of these results to clinicians managing musculoskeletal disease in patients with IBD.

Non‐inferiority was not met for the secondary outcomes in our study. Gastrointestinal surgery is a rarer outcome than IBD‐related hospitalization and the resulting wider CIs precluded a definitive determination of non‐inferiority. The fact that NSAIDs were inferior with respect to all‐cause hospitalization (lower limit of the CI above the non‐inferiority margin) was not surprising because NSAID use is associated with risk of hospitalization from several non‐IBD–related entities such as acute kidney injury and adverse cardiac events, among others. A large prospective multicenter observational study of more than 18,000 admitted patients in England found that NSAIDs were responsible for 29.6% of admissions related to adverse drug reactions, including gastrointestinal bleeding, stroke, and renal impairment.^34^ Despite the increased risk of all‐cause hospitalization, we were reassured by the lower risk of hospitalizations specifically related to IBD in the primary analysis, which was corroborated by extensive sensitivity analyses of different outcome definitions and patient subpopulations.

There are several strengths to this study. We leveraged a large cohort of patients with IBD with commercial and Medicare Advantage insurance plans across the United States, which was well‐powered to address the question of interest and enhances the generalizability of our findings to other commercially‐insured individuals in the United States. The use of IPTW to balance an extensive number of confounders associated with NSAID use optimizes the assessment of the association of NSAID exposure with IBD‐related hospitalization and helps address recent concern of residual confounding in observational studies of NSAID risk in IBD.^5^ This includes the use of validated IBD severity measures that, although not markers of disease activity per se, allowed us to better address IBD severity than previous studies. Additionally, the use of a non‐inferiority design with a margin defined by IBD content experts makes the risk assessment in this study highly clinically relevant. Finally, the consistent results across numerous sensitivity analyses adds robustness to the primary findings, including finding similar risk among the subgroup of those who repeatedly use NSAIDs and who are more likely to be using NSAIDS more consistently.

There are also several limitations that should be acknowledged. Over‐the‐counter (OTC) NSAID use cannot be ascertained in administrative claims databases. Contamination of exposure by possible OTC NSAID use in the unexposed group would bias risk estimates toward the null, but we anticipate OTC use to be low given national society guidance against NSAID use in this population and that patients with IBD are generally counseled by their gastroenterologists as such. The similar findings in the subgroup of patients exposed to COX‐2 selective inhibitors lends further confidence because there is no OTC equivalent for this NSAID subtype. Additionally, there could be channeling bias with patients exposed to prescription NSAIDs that are deemed to be of more tolerable risk by the prescribing clinician, but a multitude of risk factors were adequately balanced across groups by IPTW. Residual unmeasured confounding is also possible, including factors such as smoking and IBD disease activity that cannot be reliably measured in Optum CDM, which could bias our estimates toward or away from the null. There was longer follow‐up time in the unexposed group, but this should not significantly impact the results because HRs assess instantaneous and not cumulative risk, and the PH assumption was met, indicating that the HRs between groups were constant over time irrespective of follow‐up duration. Misclassification of outcome is possible, but the definition used is similar to previous studies in IBD,^20^, ^26^ and the concordant findings in a sensitivity analysis restricting the IBD code to the more specific primary discharge position lends reassurance. It should also be noted that although our study found non‐inferiority with respect to IBD‐related hospitalization, milder outcomes that do not necessitate inpatient care were not assessed and should be evaluated in future studies. That said, the consistent findings in a sensitivity analysis including either IBD‐related hospitalizations or ER visits at least suggest similar risk for milder flares that result in ER presentation but that are not severe enough for full inpatient admission. There is a possibility of missing data if certain diagnoses were undercoded or miscoded during insurance billing, but this issue is inherent to retrospective administrative data and missingness is expected to be nondifferential between exposure groups. Finally, although the sensitivity analysis among those with repeated NSAID use showing similar results to the primary analysis, lending reassurance to the overall findings of this study, it should be acknowledged that there is likely heterogeneity in the chronicity and extent of NSAID use among those with repeated use as defined in this study.

In conclusion, prescription NSAIDs were not associated with an increased risk of IBD‐related hospitalization beyond the predefined clinically‐acceptable margin, particularly in patients with UC. Results were consistent among patients using COX‐2 selective NSAIDs, those with repeated NSAID use, immunosuppressed patients, and among varying definitions of the primary outcome. Although further prospective study of NSAID safety in IBD is needed, the findings in this study suggest that the risk of NSAID exposure may be acceptable in select cases with strong indications for their use, such as those with significant musculoskeletal disease.

## AUTHOR CONTRIBUTIONS

All authors contributed to at least one of the following manuscript preparation roles: conceptualization AND/OR methodology, software, investigation, formal analysis, data curation, visualization, and validation AND drafting or reviewing/editing the final draft. As corresponding author, Dr Mayer confirms that all authors have provided the final approval of the version to be published and takes responsibility for the affirmations regarding article submission (eg, not under consideration by another journal), the integrity of the data presented, and the statements regarding compliance with institutional review board/Declaration of Helsinki requirements.

## Supporting information


**Disclosure Form**:

**Supplementary Table 1.** Nonsteroidal anti‐inflammatory drugs (NSAIDs) included in study.
**Supplementary Table 2.** International Classification of Diseases (ICD)‐9 and ‐10 diagnosis codes for covariates.
**Supplementary Table 3.** Current Procedural Terminology (CPT) for gastrointestinal surgical procedures.
**Supplementary Table 4.** Unadjusted risk of IBD‐related hospitalization associated with prescription NSAID use by IBD subtype.
**Supplementary Figure 1.** Overview of index date assignment for exposed and unexposed patients.
**Supplementary Figure 2.** Kernal density plots of propensity score distributions in patients with IBD (A), Crohn's disease (B), and ulcerative colitis (C).
**Supplementary Figure 3.** Covariate balance pre‐ and post‐inverse probability of treatment weighting (IPTW) in patients with IBD (A), Crohn's disease (B), and ulcerative colitis (C).
**Supplementary Figure 4.** Log‐log plots to evaluate the proportional hazards assumption in patients with IBD (A), Crohn's disease (B), and ulcerative colitis (C).
