# Supplementary material for: Safety of Prescription Nonsteroidal Anti‐inflammatory Drugs in Adults With Inflammatory Bowel Disease: Data From a Large Administrative Claims Cohort
Source: Arthritis Care Res (Hoboken). 2026 May 5;78(11):1781–90. doi: 10.1002/acr.80067 (PMC13669952; doi:10.1002/acr.80067)
Supplement: Supplementary file 2 [file ACR-78-1781-s001.docx]

**SUPPLEMENTAL MATERIAL**

**Table of Contents**

**Page 3:** Supplementary Table 1. Nonsteroidal anti-inflammatory drugs (NSAIDs) included in study: Page 2

**Page 4:** Supplementary Table 2. International Classification of Diseases (ICD)-9 and -10 diagnosis codes for covariates

**Page 9:** Supplementary Table 3. Current Procedural Terminology (CPT) for gastrointestinal surgical procedures

**Page 12:** Supplementary Table 4. Unadjusted risk of IBD-related hospitalization associated with prescription NSAID use by IBD subtype

**Page 13:** Supplementary Figure 1. Overview of index date assignment for exposed and unexposed patients.

**Page 14:** Supplementary Figure 2. Kernal density plots of propensity score distributions in patients with IBD (A), Crohn’s disease (B), and ulcerative colitis (C)

**Page 15:** Supplementary Figure 3. Covariate balance pre- and post-inverse probability of treatment weighting (IPTW) in patients with IBD (A), Crohn’s disease (B), and ulcerative colitis (C)

**Page 16:** Supplementary Figure 4. Log-log plots to evaluate the proportional hazards assumption in patients with IBD (A), Crohn’s disease (B), and ulcerative colitis (C)

**Supplementary Table 1. Nonsteroidal anti-inflammatory drugs (NSAIDs) included in study**

| Celecoxib | Indomethacin | Oxaprozin |
| --- | --- | --- |
| Diclofenac | Ketoprofen | Piroxicam |
| Diclofenac/misoprostol | Ketorolac | Rofecoxib |
| Diflunisal | Meclofenamate | Salsalate |
| Etodolac | Meloxicam | Sulindac |
| Fenoprofen | Nabumetone | Tolmentin |
| Flurbiprofen | Naproxen | Valdecoxib |
| Ibuprofen | Naproxen/sumatriptan |  |

**Supplementary Table 2. International Classification of Diseases (ICD)-9 and -10 diagnosis codes for covariates**

| Covariate | ICD-9 Codes | ICD-10 Codes |
| --- | --- | --- |
| Inflammatory arthritis (including rheumatoid arthritis, spondyloarthritis, and juvenile idiopathic arthritis) | 99.3, 696.0, 713.1, 714.xx other than 714.89-714.9x, 720.x | L40.5x, M02.3x, M05.xx, M06.0x, M06.2x, M06.3x, M06.8x, M06.9x, M07.xx, M08.xx, M45.xx, M46.1x, M46.8x-M46.9x |
| Osteoarthritis | 715.00, 715.04, 715.09-715.18, 715.20-715.28, 715.30-715.38, 715.80, 715.89-715.98, 721.0-721.3, 721.90, 721.91 | M15.xx-M19.xx |
| Other joint pain not otherwise specified (NOS) | 714.89, 714.9x | M06.4x, M12.0x-M12.1x, M12.3x, M12.8x-M12.9x, M13.0x-M13.1x, M13.8x, M14.8x, M25.4x-M25.6x, M25.8x-M25.9x, M79.6x |
| Chronic musculoskeletal pain | 307.80, 307.89, 338.0, 338.20-338.22, 338.28-338.29, 338.4x, 729.1x | F45.4x, G89.0, G89.2x, G89.4x |
| Depression | 293.83, 296.2x, 296.3x, 298.0x, 300.4, 309.0, 309.1, 309.28, 311 | F32.xx, F33.xx, F06.30, F06.31, F06.32, F34.9, F39, F43.21, F43.23 |
| Anxiety | 293.84, 300.2x, 300.0x, 308.9, 309.24, 309.89, 309.9 | F06.4, F40.xx, F41.xx, F43.0, F43.22, F43.8, F43.9 |
| Psychotic disorders | 296.0x, 296.1x, 296.4x, 296.5x, 296.6x, 296.7x, 296.8x, 296.9x, 295.xx, 297.xx, 298.xx, 780.1 | F30.xx, F31.xx, F06.33, F20.xx, F22, F23, F24, F25.xx, F28, F29, R44.xx |
| Myocardial infarction | 410.x, 412.x | I21.x, I22.x, I25.2 |
| Congestive heart failure | 398.91, 402.01, 402.11, 402.91, 404.01, 404.03, 404.11, 404.13, 404.91, 404.93, 425.4-425.9, 428.x | I09.9, I11.0, I13.0, I13.2, I25.5, I42.0, I42.5-I42.9, I43.x, I50.x, P29.0 |
| Peripheral vascular disease | 093.0, 437.3, 440.x, 441.x, 443.1-443.9, 447.1, 557.1, 557.9, V43.4 | I70.x, I71.x, I73.1, I73.8, I73.9, I77.1, I79.0, I79.2, K55.1, K55.8, K55.9, Z95.8, Z95.9 |
| Cerebrovascular disease | 362.34, 430.x-438.x | G45.x, G46.x, H34.0, I60.x-I69.x |
| Dementia | 290.x, 294.1, 331.2 | F00.x-F03.x, F05.1, G30.x, G31.1 |
| Chronic pulmonary disease | 416.8, 416.9, 490.x-505.x, 506.4, 508.1, 508.8 | I27.8, I27.9, J40.x-J47.x, J60.x-J67.x, J68.4, J70.1, J70.3 |
| Rheumatic disease | 446.5, 710.0-710.4, 714.0-714.2, 714.8, 725.x | M05.x, M06.x, M31.5, M32.x-M34.x, M35.1, M35.3, M36.0 |
| Peptic ulcer disease | 531.x-534.x | K25.x-K28.x |
| Mild liver disease | 070.22, 070.23, 070.32, 070.33, 070.44, 070.54, 070.6, 070.9, 570.x, 571.x, 573.3, 573.4, 573.8, 573.9, V42.7 | B18.x, K70.0-K70.3, K70.9, K71.3-K71.5, K71.7, K73.x, K74.x, K76.0, K76.2-K76.4, K76.8, K76.9, Z94.4 |
| Moderate or severe liver disease | 456.0-456.2, 572.2-572.8 | I85.0, I85.9, I86.4, I98.2, K70.4, K71.1, K72.1, K72.9, K76.5, K76.6, K76.7 |
| Diabetes without chronic complication | 250.0-250.3, 250.8, 250.9 | E10.0, E10.1, E10.6, E10.8, E10.9, E11.0, E11.1, E11.6, E11.8, E11.9, E12.0, E12.1, E12.6, E12.8, E12.9, E13.0, E13.1, E13.6, E13.8, E13.9, E14.0, E14.1, E14.6, E14.8, E14.9 |
| Diabetes with chronic complication | 250.4-250.7 | E10.2-E10.5, E10.7, E11.2-E11.5, E11.7, E12.2-E12.5, E12.7, E13.2-E13.5, E13.7, E14.2-E14.5, E14.7 |
| Hemiplegia or paraplegia | 334.1, 342.x, 343.x, 344.0-344.6, 344.9 | G04.1, G11.4, G80.1, G80.2, G81.x, G82.x, G83.0-G83.4, G83.9 |
| Renal disease | 403.01, 403.11, 403.91, 404.02, 404.03, 404.12, 404.13, 404.92, 404.93, 582.x, 583.0-583.7, 585.x, 586.x, 588.0, V42.0, V45.1, V56.x | I12.0, I13.1, N03.2-N03.7, N05.2-N05.7, N18.x, N19.x, N25.0, Z49.0-Z49.2, Z94.0, Z99.2 |
| Any malignancy (including lymphoma and leukemia, except malignant neoplasm of skin) | 140.x-172.x, 174.x-195.8, 200.x-208.x, 238.6 | C00.x-C26.x, C30.x-C34.x, C37.x-C41.x, C43.x, C45.x-C58.x, C60.x-C76.x, C81.x-C85.x, C88.x, C90.x-C97.x |
| Metastatic solid tumor | 196.x-199.x | C77.x-C80.x |
| AIDS/HIV | 042.x-044.x | B20.x-B22.x, B24.x |
| Gastrointestinal bleeding | 531, 531.2, 531.4, 531.6, 532, 532.2, 532.4, 532.6, 533, 533.2, 533.4, 533.6, 534, 534.2, 534.4, 534.6, 578, 578.1, 578.9, 569.3 | K92.0, K92.1, K22.10, K22.12, K22.14, K22.16, K25.0, K25.2, K25.4, K25.6, K26.0, K26.2, K26.4, K26.6, K27.0, K27.2, K27.4, K27.6, K28.0, K28.2, K28.4, K28.6, K29.0, K63.80, K31.80, K55.2, K62.5, K92.2 |
| Anal fistula | 565.1 | K60.3; K60.4 and K60.5 |
| Intestinal fistula | 569.81 | K63.2 |
| Gastrointestinal abscess | 567.22, 566, 569.5 | K65.1, K61.0, K61.1, K61.3, K63.0 |
| Intestinal stricture | 560.9, 560.89 | K56.60, K56.69 |
| Menstrual disorder | 626.xx | N91.xx, N92.xx |
| IBD severity index | See Chen G, Lissoos T, Dieyi C, Null KD. Development and Validation of an Inflammatory Bowel Disease Severity Index Using US Administrative Claims Data: A Retrospective Cohort Study. Inflamm Bowel Dis. 2021 Jul 27;27(8):1177-1183. doi: 10.1093/ibd/izaa263. PMID: 33043982; PMCID: PMC8314106. | |

**Supplementary Table 3. Current Procedural Terminology (CPT) for gastrointestinal surgical procedures**

| Surgery type | CPT code |
| --- | --- |
| Incisional and resectional procedures of bowel, intestinal transplant procedures, colon resection procedures, laparoscopic enterolysis, laparascopic and open enterostomy procedures | 44005 – 44346 |
| Open repair procedures intestine , other intestinal procedures | 44602 – 44701 |
| Open and transrectal procedures of rectum | 45000 – 45190 |
| Laparascopic and closed procedures of rectum, open repairs of rectum | 45395 – 45999 |
| Surgical incision of anus - setons and abscess drainage | 46020 – 46060 |
| Resection of anal fistula | 46270 – 46288 |
| Exploratory drainage procedures: abdomen/peritineum | 49000 – 49084 |
| Partial Colectomy | 44139, 44140, 44141, 44143, 44144, 44145, 44146, 44147, 44160, 44204, 44205, 44206, 44207, 44208, 44213 |
| Total Colectomy | 44150, 44151, 44152, 44153, 44155, 44156, 44157, 44158, 44210, 44211, 44212 |
| Ostomy (Colostomy or Ileostomy) | 44015, 44130, 44186, 44187, 44188, 44227, 44300, 44310, 44312, 44314, 44316, 44320, 44322, 44340, 44345, 44346, 45395, 45397, 44620, 44625, 44626 |
| Other fistula surgery | 44640, 44650, 44660, 44661, 45800, 45805, 45820, 45825, 46020, 46030, 46258, 46262, 46270, 46275, 46280, 46285, 46288, 46706, 46710, 46712, 46715, 46716, 46740, 46742, 57300, 57305, 57307, 57308 |
| Other abscess surgery | 10060, 10061, 10140, 10160, 10180, 45000, 45005, 45020, 46040, 46045, 46050, 46060, 49020, 49040, 49060, 49062 |
| Stricture procedures | 44615, 45150, 46700 |
| Anal/rectal repair | 44602, 44603, 44604, 44605, 44700, 44701, 45500, 45505, 45520, 45540, 45541, 45550, 45560, 45562, 45563, 45900, 45905, 45910, 45915 |
| Enterectomy | 44120, 44121, 44125, 44126, 44127, 44128, 44202, 44203 |
| Proctectomy | 45110, 45111, 45112, 45113, 45114, 45116, 45119, 45120, 45121, 45123, 45126 |
| Other (exploratory laparotomy and lysis of adhesions) | 44005, 49000 |
| Small Bowel Resection | 44202, 44203, 44120, 44130, 44186, 44187 |

**Supplementary Table 4. Unadjusted risk of IBD-related hospitalization associated with prescription NSAID use by IBD subtype**

| IBD subtype | Unadjusted HR (95% CI) |
| --- | --- |
| All IBD | 1.22 (1.19-1.26) |
| Crohn’s disease | 1.37 (1.32-1.42) |
| Ulcerative colitis | 1.10 (1.05-1.15) |

**Supplementary Figure 1. Overview of index date assignment for exposed and unexposed patients.**

*Legend: mo: month; IBD: inflammatory bowel disease.*

**Supplementary Figure 2.** **Kernal density plots of propensity score distributions in patients with IBD (A), Crohn’s disease (B), and ulcerative colitis (C)**

**Supplementary** **Figure 3. Covariate balance pre- and post-inverse probability of treatment weighting (IPTW) in patients with IBD (A), Crohn’s disease (B), and ulcerative colitis (C)**

**Supplementary Figure 4. Log-log plots to evaluate the proportional hazards assumption in patients with IBD (A), Crohn’s disease (B), and ulcerative colitis (C)**
